# Effects of Microbial Inoculants from Three Nutrient-Poor Environments on Soil Improvement and Plant Growth Promotion in Sandy Soil

**DOI:** 10.3390/microorganisms14030722

**Published:** 2026-03-23

**Authors:** Xin Sun, Xuanran Yu, Xingyu Zhang, Xinxin Yang, Rengui Xue, Aodeng Rong, Xin Liu, Xiongfei Zhang, Chong Li, Jinchi Zhang

**Affiliations:** 1 Co-Innovation Center for Sustainable Forestry in Southern China of Jiangsu Province, Key Laboratory of Soil and Water Conservation and Ecological Restoration of Jiangsu Province, Nanjing Forestry University, Nanjing 210037, Chinayuxuanran@njfu.edu.cn (X.Y.);; 2 Administration and Protection Center of Songshushan Nature Reserve in Inner Mongolia, Songshushan Forest Farm of Wengniute Banner, Chifeng 024500, China; 3 Inner Mongolia Big Data Center, Hohhot 010090, China; 4 Department of Renewable Resources, University of Alberta, Edmonton, AB T6G 2E3, Canada

**Keywords:** microbial inoculants, sandy soil, saline-alkali soil, mining area

## Abstract

Approximately 20% of China’s land area is desertified or highly desertifiable, where loose sandy soil and low nutrient availability restrict plant growth. Microbial inoculants, as an emerging ecological restoration technology, play a key role in plant growth and soil nutrient activation in sandy regions. However, a systematic understanding of functional differences among microorganisms isolated from different stressed environments remains insufficient. Nine functional microbial strains from three stressed habitats, including sandy land, coastal saline-alkali soil, and heavy metal mining areas, were selected to conduct a three-month pot experiment, investigating their effects on soil nutrient activation, plant growth and microbial communities. Results showed that all inoculants increase plant biomass (by 4.15~25.59%), with KS-33, KS-36, SD-13 and SD-3 significantly promoting biomass in different plant parts (*p* < 0.05), and with YJ-15 remarkably enhancing root growth (root length increased by 70.83%, *p* < 0.01). Inoculation reduced bacterial Chao1 by 27.18~53.97%, but increased fungal Chao1 by 12.77~28.38% (except SD-30). Bacterial generalist species proportion increased from 61.12% to 83.78~93.99% after inoculation, higher than the variation degree of the fungal community. Mantel analysis revealed a reverse trend between soil nutrients, water content and plant growth. This may be associated with the increased consumption by plants and microorganisms. In summary, microbial inoculants enhance nutrient cycling processes and plant growth by reshaping soil microbial communities. Performance of microbial inoculants is more likely governed by their inherent ecological functions rather than being entirely determined by their original environments. Despite varying mechanisms, these inoculants can effectively enhance sandy soil microbial communities, providing a theoretical basis for regional ecological restoration.

## 1. Introduction

At present, land desertification has become a global environmental issue. The global average area of deserts is approximately 17.64 × 10^6^ square kilometers, accounting for 12% of the Earth’s land area [1]. According to the report of the United Nations Conference to Combat Desertification [2], approximately 75% of the world’s arid lands are threatened by desertification. The *2024 China Ecological and Environmental Status Bulletin* indicates that desertified land and extremely desertification-prone land in China account for approximately 20% of the country’s total land area. Desertification is attributed to anomalous water cycles induced by global warming and extreme weather events [3]. Moreover, urbanization, unsustainable farming practices, the degradation of surface vegetation and other human-induced factors cannot be ignored as well [4,5,6,7]. Desertified land is often characterized by low soil nutrient content, loose soil structure, poor water and nutrient retention capacity, and low microbial activity [8,9,10], which severely restricts the natural restoration process of surface vegetation and the development of regional economies [11]. To release the ecological impacts of desertification on regional ecosystems, artificial remediation measures have been widely adopted. Methods such as straw checkerboard barriers can restore the environment and enhance the stability of microbial community structure in sandy soil [12]. However, they suffer from drawbacks including long time consumption and high potential maintenance costs. Sand fixation using drought-tolerant plants can gradually accumulate and improve regional ecological conditions, but it suffers from a long time lag for effectiveness and high maintenance expenses. In contrast, microbial inoculants have emerged as one of the promising ecological remediation approaches in recent years [13,14,15,16]. They can activate native soil microbial communities [17,18,19], dissolve mineral particles to release available nutrients [20,21], and facilitate the formation of soil microaggregates. Furthermore, they can promote vegetation growth by creating favorable soil microenvironments [22]. Microorganisms have been proven to play a pivotal role in regulating material cycling and improving regional ecosystems. However, do dominant strains from different adverse sites share similarities in soil nutrient release and plant growth promotion [23]? What differences exist in the impacts of dominant strains of different origins on the native microbial community in sandy land? Our understanding of the performance of strains from different sources in sandy land soils remains limited. Therefore, nine microbial inoculant treatments were set up in this study, namely KS-32, KS-33 and KS-36 isolated from heavy metal mines, SD-13, SD-30 and SD-33 screened from the Horqin Sandy Land (the study area), and YJ-12, YJ-15 and YJ-22 obtained from coastal saline-alkali soils. The study investigated their effects and differences on soil nutrients, plant growth and microbial communities in sandy land. Taking aeolian sandy soil of the Horqin Sandy Land as the research object, this study selected alfalfa (*Medicago sativa* L. cv. Aohan) as the test plant. The hypotheses of this study are as follows: (1) All the microorganisms isolated from different adverse sites can improve soil physical and chemical properties and promote plant growth in sandy soils. (2) The inoculation effects differ from each other significantly and are not entirely consistent with their original regions.

## 2. Materials and Methods

### 2.1. Experimental Materials

#### 2.1.1. Microbial Inoculants

Soil samples were randomly collected from three regions and placed in sterilized plastic bags, which were sealed and transported back to the laboratory. Bacteria in the soil were isolated using the gradient dilution method. According to the method described by Shi [24], NBRIP medium was used to detect the phosphate-solubilizing ability of the bacteria [25]; nitrogen-fixing ability was tested using Ashby’s nitrogen-free medium [26]; the production of indole-3-acetic acid (IAA) was determined by the Salkowski colorimetric method [27]. Finally, nine strains were chosen and preserved in the laboratory.

The genomic DNA of the isolated strains was extracted, and the 16S rRNA gene was amplified with universal primers 27F (5′-AGAGTTTGATCCTGGCTCAG-3′) and 1492R (5′-GGTTACCTTGTTACGACTT-3′) in 50 μL PCR reactions containing 2× Taq MasterMix, primers and template DNA, and products were verified on 1% agarose gels. The PCR products were sequenced on an ABI3730 sequencer by Sanger method, and the obtained sequences were compared with the NCBI nt database by BLAST+ (v2.16.0) for species annotation. The sequencing quality was controlled by checking the signal normality and splicing miscellaneous peaks; all 9 strains had normal sequencing signals. The strains were identified as: *Priestia aryabhattai* YJ-22, *Priestia megaterium* YJ-15 and *Rossellomorea aquimaris* YJ-12, isolated from the coastal saline-alkali soil in Dafeng District, Yancheng City, Jiangsu Province, China (120°45′36″ E, 33°02′24″ N); *Priestia aryabhattai* SD-30, *Bacillus* sp. SD-13 and *Priestia megaterium* SD-33, isolated from the aeolian sandy soil at the western edge of the Horqin Sandy Land in Ongniud Qi, Chifeng City, Inner Mongolia Autonomous Region, China (119°00′36″ E, 42°56′24″ N); and *Pseudomonas* sp. KS-33, *Bacillus safensis.* KS-32 and *Pseudomonas chlororaphis* KS-36, isolated from the soil of an abandoned chromite mine in Ma’anshan City, Anhui Province, China (118°30′36″ E, 31°40′12″ N). Nine strains were individually cultured on LB (Luria–Bertani) solid medium at 28 °C for 24 h. A single colony was then picked and inoculated into LB liquid medium, followed by shake cultivation in a shaker incubator at 28 °C for 48 h to complete the preparation. The concentration was adjusted to 1.0 × 10^8^ CFU/mL with sterile water, and an equal volume of sterilized LB liquid medium was added to the control group.

#### 2.1.2. Experimental Plant

*Medicago sativa* L. cv. Aohan was used as the experiment plant, which is characterized by cold and drought tolerance as well as strong stress resistance, and is thus suitable for cultivation in Chifeng, Inner Mongolia. Prior to germination, the seeds were placed in a beaker, rinsed thoroughly with warm water at 60 °C with constant stirring, then soaked in cold water for 24 h, and finally rinsed with distilled water for subsequent use.

#### 2.1.3. Experimental Soil

The experiment soil was collected from the western edge of the Horqin Sandy Land in Chifeng City, Inner Mongolia Autonomous Region, where a mid-temperate semi-arid continental monsoon climate prevails with an annual precipitation of 300–450 mm concentrated in summer. This region is characterized by dry climate, frequent aeolian sand activities, aeolian sand-dominated soil, and sparse vegetation mainly consisting of open forest steppe and meadow. Prior to the pot experiment, stones and root residues were removed from the soil, which was then thoroughly homogenized for subsequent use.

### 2.2. Experiment Design

This study was conducted from April to July 2025 for a duration of three months in a constant temperature incubator under the following set conditions: temperature of 25 °C and a photoperiod of 8 h per day. In accordance with the rainfall conditions in Inner Mongolia, a fixed volume of 30 mL of water was irrigated per pot per day. We use ultrapure water for irrigation to reduce the effects of miscellaneous bacteria, other ions, or impurities on the soil microbial community structure. The plastic pots used for the pot experiment had a bottom diameter of 9 cm, an opening diameter of 11.8 cm and a height of 12.7 cm. A total of nine treatment groups were set up in the experiment with three replicates per group, amounting to 30 pots in all. Twenty seeds were sown per pot, and seedlings were thinned to maintain 10 plants per pot. Five plants were randomly selected from each pot for subsequent determination. Microbial inoculation was performed once at the root system after seed germination. Each treatment group was inoculated with 20 mL of the functional microbial inoculant per pot. An equal volume of sterilized LB liquid medium was added to the control group. Soil and plant samples were separately collected in July 2025 for subsequent index determination.

### 2.3. Sample Collection

Five plants were selected from each treatment group for sampling randomly. Soil was removed from the root system, and the whole plants were rinsed thoroughly. After removing excess water, plant height and root length were measured. The aboveground and underground parts of the plants were weighed separately, placed in paper envelopes, subjected to de-greening treatment at 105 °C for 30 min, and then dried at 85 °C to a constant weight.

For soil sampling, three soil samples were collected from each treatment group. Air-dried soil samples were passed through a 2 mm sieve to remove impurities, while the other two samples were stored in sterile sealed bags at −80 °C, which were used for the determination of basic soil physical and chemical properties and the assessment of soil microbial characteristics, respectively.

### 2.4. Sample Determination

#### 2.4.1. Determination of Soil Physical and Chemical Properties

Soil pH was determined by potentiometric measurement with a pH meter (PHSJ-3F, IMSEA Scientific Instrument Co., Ltd., Shanghai, China) [28]. Electrical conductivity was measured using a conductivity meter (DDS-307A, IMSEA Scientific Instrument Co., Ltd.). Soil available phosphorus was extracted and determined via the hydrochloric acid–sulfuric acid extraction method [29], available potassium was extracted with ammonium acetate and measured by a flame photometer (AA-7000, Shimadzu Corporation, Kyoto, Japan) [28], and soil ammonium nitrogen was determined by the KCl extraction–indophenol blue colorimetric method. The total carbon and nitrogen in the soil were analyzed using an elemental analyzer (vario EL III, Elementar Analysensysteme GmbH, Langenselbold, Germany). Soil ammonium nitrogen and nitrate nitrogen were determined using potassium sulfate extraction, followed by measurement with a continuous flow analyzer (SAKLAR SAN++/S-011300110537) [30].

#### 2.4.2. DNA Extraction and Illumina Sequencing

Total genomic DNA was extracted from 0.5 g rhizospheric soil samples using the E.Z.N.A.^®^ Soil DNA Kit (Omega Bio-tek, Norcross, GA, USA) following the manufacturer’s protocol. DNA integrity was verified by 1% agarose gel electrophoresis, and concentration and purity were determined with a NanoDrop 2000 spectrophotometer (Thermo Fisher Scientific (China) Co., Ltd., Shanghai, China).

For PCR amplification: The bacterial 16S rRNA gene V3–V4 region was amplified using the primer pair 338F (5′-ACTCCTACGGGAGGCAGCAG-3′) and 806R (5′-GGACTACHVGGGTWTCTAAT-3′) [31]. The fungal ITS1 region was targeted with the primer pair ITS1F (5′-CTTGGTCATTTAGAGGAAGTAA-3′) and ITS2R (5′-GCTGCGTTCTTCATCGATGC-3′) [32]. All PCR reactions were performed in a 20 μL system containing 10 μL of 2× Pro Taq HS Premix (Accurate Biology, Changsha, China; including Taq DNA polymerase, dNTPs, and reaction buffer), 0.8 μL of each forward/reverse primer (10 μM), 1 μL of template DNA (50–100 ng), and 7.4 μL of sterile double-distilled water. Amplification was carried out on an ABI GeneAmp^®^ 9700 thermal cycler (Applied Biosystems, Foster City, CA, USA) with the following conditions: initial denaturation at 95 °C for 3 min; followed by 27 cycles (for 16S rRNA gene) or 35 cycles (for ITS1 region) of denaturation at 95 °C for 30 s, annealing at 55 °C for 30 s, and extension at 72 °C for 45 s; and a final extension at 72 °C for 10 min.

Amplicons were purified from 2% agarose gels with the AxyPrep DNA Gel Extraction Kit (Axygen Biosciences, Union City, CA, USA), pooled at equimolar concentrations, and paired-end sequenced (PE250) on an Illumina platform by Shanghai Majorbio Bio-pharm Technology Co., Ltd. (Shanghai, China) All raw data were deposited in NCBI SRA under accession number PRJNA1412535 and PRJNA1412548.

### 2.5. Data Analysis

The resulting sequences were quality filtered with fastp (https://github.com/OpenGene/fastp, v0.23.4, accessed on 1 December 2025) and merged with FLASH (https://ccb.jhu.edu/software/FLASH/index.shtml, v1.2.11, accessed on 1 December 2025) [33]. Then the high-quality sequences were de-noised using DADA2 [34] plugin in the Qiime2 [35] pipeline with recommended parameters, which obtains single nucleotide resolution based on error profiles within samples. DADA2 denoised sequences are usually called amplicon sequence variants (ASVs). To mitigate the effects of sequencing depth variation on α and β diversity analyses, ASVs affiliated with o_chloroplasts and f_mitochondria were discarded. Subsequently, all samples were rarefied to an equal sequencing depth of 33,815 sequences per sample based on the minimum library size using the USEARCH v11. This rarefaction process generated a total of 8401 valid ASVs. Rarefaction represents a classic normalization strategy for microbial α diversity analysis, which effectively eliminates biases in species richness estimation induced by unequal library sizes and complies with the best practices in microbial ecology research. Taxonomic assignment of ASVs was performed using the Naive bayes consensus taxonomy classifier implemented in Qiime2 and the SILVA 16S rRNA database (v138).

Levin’s niche breadth analysis [36,37]:

$$B_{i}=\frac{1}{\sum_{j=1}^{n}P_{ij}^{2}}$$

n:sample size; $P_{ij}:$ *the abundance of species j in sample i; the value of Bi ranges from 1 to n; the larger the value, the wider the ecological niche.*

The niche width was calculated using the spaa package (v 0.2.2) in R 3.3.1, and species were classified accordingly. Species with a high niche width (>2) were defined as generalists or ubiquitous species, while those with a low niche width (<1.5) were considered specialists or specialized species. The proportions of these groups were compared; a higher proportion of generalist species indicates weaker environmental sensitivity.

## 3. Results

### 3.1. Effects of Microbial Inoculants on Plant Growth

**Figure 1:**
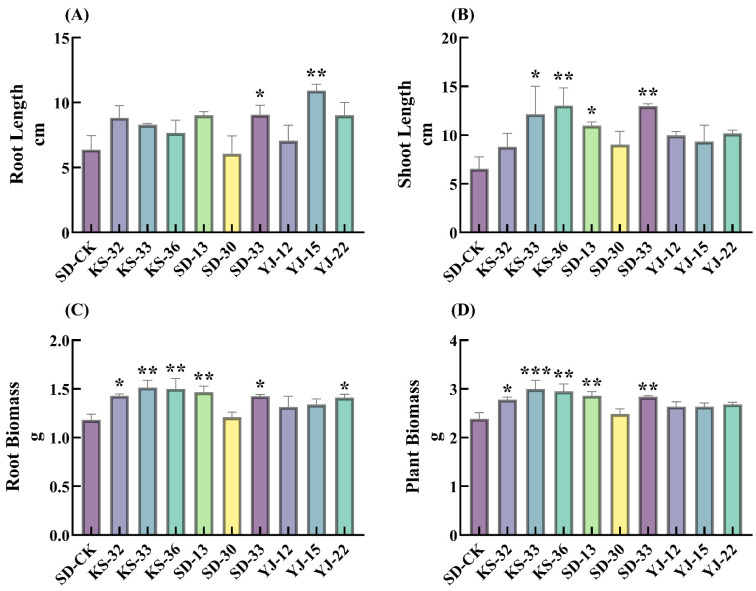
The effect of different microbial inoculant treatments on plants. (**A**) Root Length, cm. (**B**) Shoot Length, cm. X-axis represents different types of microbial inoculants treatments. (**C**) Root biomass, g. (**D**) Plant biomass, g. One-way analysis of variance (ANOVA) was used to analyze significant differences between different treatment groups and the control group (SD-CK). * indicates *p* < 0.05; ** indicates *p* < 0.01; *** indicates *p* < 0.001.

A comparative analysis of the control and treatment groups revealed that the vast majority of the screened microbial inoculants significantly improved total biomass (F_(9,20)_ = 3.526, *p* = 0.009), root length (F_(9,20)_ = 2.657, *p* = 0.033) and root biomass (F_(9,20)_ = 3.020, *p* = 0.019) in sandy soil. KS-36, KS-33, SD-13 and SD-33 showed a tendency to increase plant height (F_(9,20)_ =2.063, *p* = 0.085) and total biomass (Figure 1C,D). Under the treatments of KS-36 and KS-33, plant height was increased by 26.87% (*p* < 0.05) and 28.16% (*p* < 0.01), respectively (Figure 1B), while SD-13 and SD-33 enhanced plant biomass by 19.80% (*p* < 0.01) and 18.94% (*p* < 0.01), respectively. In contrast, YJ-15 and KS-32 mainly focused on promoting plant root growth: YJ-15 significantly increased underground root length (by 70.83%, *p* < 0.01), and KS-32 markedly elevated the root biomass (by 68.32%, *p* < 0.05). Although most strains exhibited plant growth-promoting effects, there were still differences in the specific plant parts they promoted.

### 3.2. Effects of Microbial Inoculants on Soil Quality

**Figure 2:**
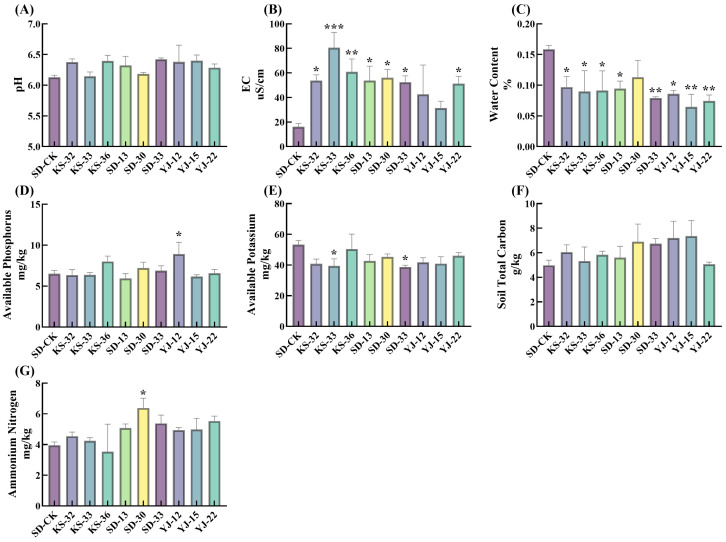
The effect of different microbial inoculant treatments on soil. (**A**) pH. (**B**) Soil electrical conductivity, uS/cm. (**C**) Water Content, %. (**D**) Available Phosphorus, mg/kg. (**E**) Available Potassium, mg/kg. (**F**) Soil Total Carbon, g/kg. (**G**) Ammonium Nitrogen X-axis represents different types of microbial inoculant treatments. One-way analysis of variance (ANOVA) was used to analyze significant differences between different treatment groups and the control group (SD-CK). * indicates *p* < 0.05; ** indicates *p* < 0.01; *** indicates *p* < 0.001.

Microbial inoculants showed a tendency in increasing the pH (F_(9,20)_ = 0.985, *p* = 0.482) (Figure 2A), total carbon content (F_(9,20)_ = 0.922, *p* = 0.527) (Figure 2F) and electrical conductivity (F_(9,20)_ = 2.579, *p* = 0.037) of sandy soil, with the soil electrical conductivity being significantly elevated by 94.17–397.59% (Figure 2B). In contrast, soil water content and available potassium content were significantly reduced (Figure 2C,E). A negative correlation trend was observed between plant growth and soil water content as well as available nutrients after inoculant application. Under the KS-33 treatment, plant height was increased by 28.16% (*p* < 0.01) and biomass by 165.25% (*p* < 0.001), while available potassium was decreased by 35.05% (*p* < 0.05) and soil water content by 77.24% (*p* < 0.05) (Figure 1 and Figure 2C–E). In sandy soil, only YJ-12 significantly increased soil available phosphorus content (by 36.92%, *p* < 0.05) (Figure 2D), which might be attributed to its phosphorus-solubilizing characteristic. There was no significant change in soil water content under the SD-30 treatment, which was generally consistent with its previously observed trends in root growth promotion (Figure 1) and soil available nutrient activation (Figure 2D,E). Furthermore, microbial inoculants enhanced the ammonium nitrogen concentration in sandy soil, and inoculation with strain SD-30 resulted in a significant increase of 61.52% (Figure 2G).

### 3.3. Effects of Microbial Inoculants on the Structure of Soil Microbial Community

**Figure 3:**
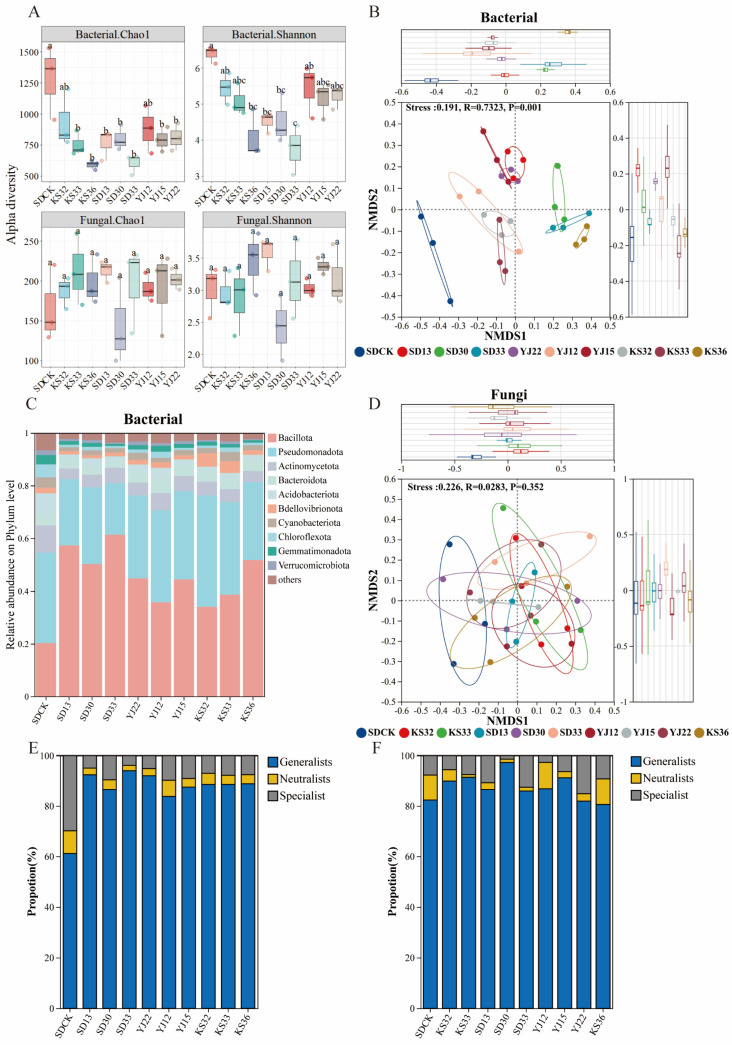
Effects of different microbial inoculant treatments on soil microbial community. (**A**) Microbial Alpha diversity, including the Chao1 index and Shannon index of bacteria and fungi. (**B**) NMDS analysis shows bacteria Beta diversity under different inoculant treatments. (**C**) Stacked bar chart of the relative abundance of microbial phyla under different treatments. (**D**) NMDS analysis shows fungi Beta diversity under different inoculant treatments. (**E**) Proportions of Generalists species, Specialists species, and Neutralists species in the bacterial community. (**F**) Proportions of generalist species, specialist species, and neutralist species in the fungi community. One-way analysis of variance (ANOVA) was used to analyze significant differences in different groups. Different letters indicate significant differences among different treatments.

All microbial inoculants decreased the soil bacterial Chao1 (F_(9,20)_ = 4.591, *p* = 0.002) and Shannon (F_(9,20)_ = 6.106, *p* = 0.001) indices (*p* < 0.05) (Figure 3A), but increased the soil fungal Alpha diversity to a certain extent (Chao1: F_(9,20)_ = 0.896, *p* = 0.546; Shannon: F_(9,20)_ = 1.881, *p* = 0.115) (*p* > 0.05). Among them, KS-32 and YJ-12 exhibited the minimal changes in Alpha diversity: the Chao1 index was reduced by 37.34% (*p* < 0.05) and 46.20% (*p* < 0.05), respectively; the Shannon index was decreased by 17.48% (*p* < 0.05) in both treatments, while the fungal Chao1 index was increased by 12.77% (*p* > 0.05) and 14.99% (*p* > 0.05); NMDS analysis based on Bray–Curtis distances revealed that inoculant application significantly altered the soil bacterial community structure (Stress = 0.191, *p* = 0.001) (Figure 3B), whereas it had a slight effect on the fungal community structure (Figure 3D). Notably, the soil bacterial and fungal community structures after inoculation with KS-32, KS-33 and YJ-12 were relatively similar. Analysis of changes in the relative phylum abundance showed that all microbial inoculant treatments significantly increased the relative abundance of *Bacillota* in the soil, while the relative abundances of native soil microbial phyla with low initial abundances such as *Gemmatimonadota* and *Chloroflexota* were further reduced (Figure 3C). Fungi were also affected by the inoculant (Figure S1). The addition of the inoculant decreased the relative abundance of the indigenous dominant phylum *Ascomycota*, while increasing the relative abundance of other phyla. Among them, functional microorganisms screened from sandy soil had a minor effect on the soil fungal community structure.

Analysis of soil microbial niche breadth indicated that the proportion of generalists in the bacterial community increased from 61.12% to 83.78–93.99%, and the proportion of specialists decreased from 29.89% to 3.89–9.79% (Figure 3E). In the fungal community of the control group, the proportions of generalists and specialists were 82.36% and 7.72%, respectively; in the treatment groups, the proportion of generalists ranged from 80.57% to 97.26% and that of specialists from 1.41% to 15.12%, showing no significant differences from the control group. Therefore, the variation degree of the bacterial community was more significant than that of the fungal community.

### 3.4. Responses of the “Microbial Community–Soil–Plant” System to Microbial Inoculants

Microbial inoculants significantly altered the soil microbial community, and the changes in the relative abundance of each phylum as well as the shifts in community structure within the microbial community were likely closely associated with soil physical and chemical properties and plant growth. On the one hand, inoculants improved soil properties and enhanced plant growth by increasing the relative abundance of specific phyla in the soil; for instance, microbial inoculant treatments increased the relative abundance of *Firmicutes* in the soil, accompanied by significant increases in plant height (*p* < 0.05) and aboveground dry weight (*p* < 0.05). On the other hand, microbial inoculants promoted an increase in plant biomass by reducing the relative abundance of specific microbial phyla such as *Chloroflexota*, which was significantly negatively correlated with aboveground dry weight (*p* < 0.01).

**Figure 4:**
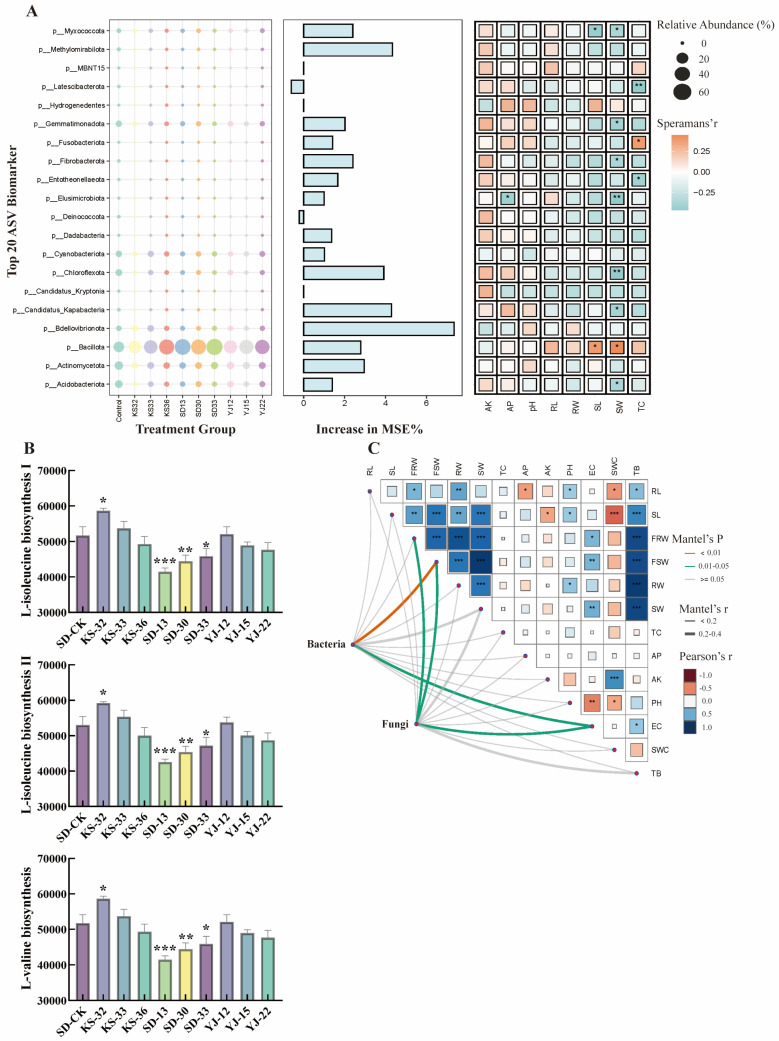
Impacts of Variations in Different Microbial Communities on Soil Nutrients and Plant Growth. (**A**) Impacts of variations in bacterial relative abundance at phylum level on plant growth and soil nutrients and prediction of their relative importance. The ordinate represents the top 20 bacterial phyla by relative abundance. The abscissa on the left denotes different treatment groups, and the variation in bubble size indicates the change in the relative abundance of microbial communities under each treatment group. A random forest model was constructed in the middle panel to predict the relative importance of each bacterial phylum. The right panel shows the correlations between the relative changes in different bacterial phyla and soil nutrients as well as plant growth parameters. The correlations were expressed using Spearman’s correlation coefficient. (**B**) Changes of MetaCyc metabolic pathways (L-Isoleucine biosynthesis I/II, L-valine biosynthesis) inferred by PICRUSt2. (**C**) Mantel analysis of correlations between fungal/bacterial community structures and soil–plant indicators; Pearson coefficients denote soil–plant relationships, with colored lines and * indicating significant correlations. * indicates *p* < 0.05; ** indicates *p* < 0.01; *** indicates *p* < 0.001. Abbreviations denote different indicators: RL = Root Length; SL = Shoot Length; FRW = Fresh Root Weight; FSW = Fresh Shoot Weight; RW = Root Weight; SW = Shoot Weight; TC = Total Carbon; AK = Available Potassium; AP = Available Phosphorus; EC = Electrical Conductivity; SWC = Soil Water Content; TB = Total Biomass.

Aboveground biomass during plant growth was more closely correlated with the relative abundance of soil microorganisms (Figure 4A). Mantel analysis further revealed that changes in the overall structures of bacterial and fungal communities also had significant effects on soil nutrients and plant growth (Figure 4B). Shifts in bacterial and fungal community structures additionally exerted a significant influence on plant biomass and soil electrical conductivity (EC). Notably, plant growth was negatively correlated with soil available nutrients: root length was negatively correlated with available phosphorus (*p* < 0.05); plant height was negatively correlated with available potassium (*p* < 0.05); and soil water content was significantly negatively correlated with plant growth. Functional information of MetaCyc pathways was obtained via PICRUSt2 analysis, and the results of ANOVA and Tukey–Kramer tests showed that the KS-32 treatment may increase the abundance of branched-chain amino acid synthesis pathways in soil microorganisms, with 13.38% (*p* < 0.05) and 11.78% (*p* < 0.05) increases in L-isoleucine biosynthesis I and L-isoleucine biosynthesis II, respectively, and a 13.38% (*p* < 0.05) increase in L-valine biosynthesis. Similar functional trends were also observed in the YJ-12 and KS-33 treatments.

## 4. Discussion

Microbial inoculants significantly alter the diversity and community structure of soil microorganisms, thereby influencing soil nutrient cycling processes and regulating plant growth [38,39,40]. Researchers suggest that *Bacillota* was significantly positively correlated with processes such as amino acid synthesis [41]. KS-32 may enhance the activity of amino acid-related pathways by increasing its relative abundance, a result that is also supported by functional prediction using PICRUSt2. We also observed a decrease in the relative abundance of *Acidobacteria*, which may be attributed to its generally recognized low competitive ability in resource-rich soils [42,43]. Several studies have found that microbial inoculants may recruit beneficial microbial communities by influencing root exudates [18,44,45]. In this study, all the screened microbial inoculants promoted plant growth and improved soil nutrient levels by expanding the niche breadth of plant-growth-promoting bacteria. Numerous studies have reported that the application of microbial inoculants reduced soil microbial α-diversity [46], which is consistent with the conclusions of our study. Notably, microbial inoculants exert a significant effect on the structure of soil bacterial and fungal communities, even in cases of inoculation failure [47]—that is, even when plant growth and soil nutrients are not significantly affected, the inoculation still markedly alters the niche breadth of the soil microbial community [48]. In our study, we found that the increases in plant height, root length and biomass were more closely associated with changes in the soil microbial community structure. However, the potential negative risks associated with the application of microbial inoculants also merit attention. A decline in α-diversity may lead to a reduction in soil multifunctionality and nutrient loss [49]. Recent research by Wagg [50] has also indicated that soil microbial α-diversity is closely linked to the stability of ecosystem functions; its enhancement can synchronously improve the temporal stability of multiple ecosystem functions. Specifically, the higher the stability of microbial taxa that support specific ecosystem functions, the higher the stability of those functions themselves. In addition, a significant increase in soil electrical conductivity may also cause certain damage to water cycling and soil structure [51]. Under such conditions, how long the homogenization caused by the significant reduction in microbial diversity will persist, and how to strike a balance between short-term plant growth promotion and long-term regional ecological stability, remains one of the key issues in current research.

Numerous studies have shown that microbial inoculants can significantly increase soil nutrients [52,53], but this effect was not obvious in the present study. The decrease in soil water content may also be attributed to the enhanced metabolic activities of plants and microorganisms. Relevant studies have also suggested that the stress resistance of plants to water deficit might thus be enhanced [54,55]. However, we did not conduct a time-series analysis and only measured nutrient and water changes once, so there are still certain limitations.

KS-32, KS-33 and YJ-12 all likely enhance nutrient cycling processes such as nitrogen turnover by altering the relative abundance and community structure of certain soil microorganisms, thereby further influencing plant growth and soil nutrient acquisition. This is consistent with the findings of Jia and Nie [13,36]. In addition, most microbial inoculants isolated from the same source exhibited consistent effects on the structure of soil bacterial communities after inoculation; for example, YJ-22 and YJ-15 showed extremely high similarities in plant growth promotion, nutrient activation and soil microbial community structure. However, not all microbial inoculants from different sources presented significant differences. For instance, the reconstructed bacterial community structures of KS-32, YJ-12 and KS-33 in the NMDS analysis were basically consistent. Interestingly, the functional microorganisms isolated from mining and saline-alkali areas performed no worse in sandy soil than SD-33, a strain screened from sandy soil itself. It is thus evident that among microbial communities, significant differences still exist between microbial inoculants isolated from different adverse site conditions, yet cross-site inoculation does not equate to reduced efficacy. Functional microorganisms with convergent functions are also present in different adverse environments, and they exhibit striking consistency in regulating plant growth and soil nutrient cycling processes. However, this study only focuses on a single strain and does not involve research on synthetic microbial consortia [56]. The construction of synthetic consortia under different environmental conditions will be the focus of our subsequent research.

However, our analysis of the metabolic processes was based on predictive results from PICRUSt2, and lacks validation by metagenomics and transcriptomics. Therefore, in future research, we will supplement the dynamic monitoring of nutrient turnover processes, conduct precise quantification using metagenomics, and extend the monitoring period to investigate the changes in soil microbial communities following inoculation, as well as the restoration process. Furthermore, in future research, we will adopt a time-series sampling strategy and delve into the microscopic level to investigate the root colonization dynamics of the inoculant, thereby obtaining more comprehensive and informative data.

## 5. Conclusions

This study demonstrates that microbial inoculants from different adverse habitats can effectively improve sandy soil. The inoculants significantly reshaped the soil microbial community structure. It has a particularly prominent regulatory effect on the bacterial community and an increased proportion of generalist species in the bacterial community. Their core growth-promoting mechanism lies in expanding the ecological niches of dominant bacterial phyla and enhancing the functional contribution of generalist taxa. Some strains may promote plant nitrogen turnover by regulating amino acid synthesis pathways. Inoculation significantly improved plant growth indicators, and indirectly regulated soil physicochemical properties through microbe–plant interactions, resulting in increased soil electrical conductivity and decreased soil water content.

## Data Availability

All data are provided in this article, and the bacterial and fungal sequencing data have been deposited in the NCBI database. All raw data were deposited in the NCBI Sequence Read Archive (SRA) under the accession numbers PRJNA1412535 and PRJNA1412548.

## Supplementary Materials

The following supporting information can be downloaded at https://www.mdpi.com/article/10.3390/microorganisms14030722/s1, Figure S1: Stacked bar chart of the relative abundance of microbial phyla under different treatments.
