# Supplementary material for: Effects of Microbial Inoculants from Three Nutrient-Poor Environments on Soil Improvement and Plant Growth Promotion in Sandy Soil
Source: Microorganisms. 2026 Mar 23;14(3):722. doi: 10.3390/microorganisms14030722 (PMC13029691; doi:10.3390/microorganisms14030722)
Supplement: Supplementary file 1 [file microorganisms-14-00722-s001.zip › microorganisms-4144463-supplementary.pdf]

Effects of microbial inoculants from three nutrient-poor environments on soil improvement and plant growth promotion in Sandy soil

Xin Sun [a,#], Xuanran Yu [a,#], Xinyu Zhang [a], Xinxin Yang [b], Rengui Xue [a], Aodeng Rong [c], Xin Liu [a], Xiongfei Zhang [a], Chong Li [d], [a] and * Jinchi Zhang

[a] Co-Innovation Center for Sustainable Forestry in Southern China of Jiangsu Province, Key Laboratory of Soil and Water Conservation and Ecological Restoration of Jiangsu Province, Nanjing Forestry University, Nanjing 210037, China.

[b] Administration and Protection Center of Songshushan Nature Reserve in Inner Mongolia, Songshushan Forest Farm of Wengniute Banner, Chifeng, Inner Mongolia 024500, China.

[c] Inner Mongolia Big Data Center, Hohhot 010090, China.

[d] Department of Renewable Resources, University of Alberta, Edmonton, AB T6G 2E3, Canada.

#These authors contributed equally to this work.

* Correspondence: Author: Jinchi Zhang (zhang8811@njfu.edu.cn). **ORCID:** Xuanran Yu (0009-0004-4296-3355); Chong Li (0000-0001-9330-5396); Xin Liu (0000-0001-8641-7170); Xiongfei Zhang: (0009-0004-9285-9038); Rengui Xue (0009-0002-1674-3530); Jinchi Zhang (0000-0002-0517-7214).

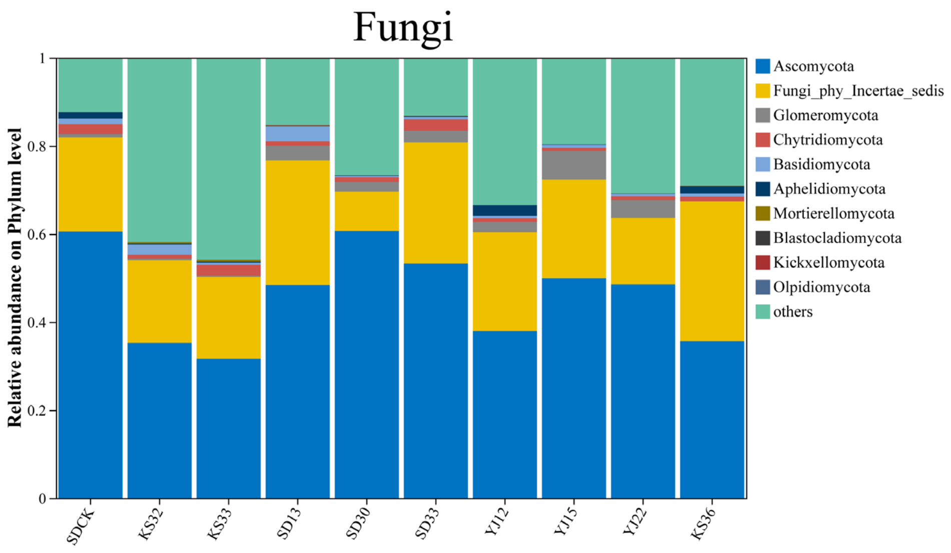

Figure.S1. Stacked bar chart of the relative abundance of microbial phyla under different treatments.
